# Management of face presentation, face and lip edema in a primary healthcare facility case report, Mbengwi, Cameroon

**DOI:** 10.11604/pamj.2019.33.292.18927

**Published:** 2019-08-08

**Authors:** Nzozone Henry Fomukong, Ngouagna Edwin, Mandeng Ma Linwa Edgar, Ngwayu Claude Nkfusai, Yunga Patience Ijang, Fala Bede, Joyce Shirinde, Samuel Nambile Cumber

**Affiliations:** 1 Microhealth Global Medical Centre, Mbengwi, Cameroon; 2 Department of Medicine and Surgery, Faculty of Health Sciences University of Buea, Buea, Cameroon; 3 Department of Microbiology and Parasitology, Faculty of Science, University of Buea, Buea, Cameroon; 4 Cameroon Baptist Convention Health Services (CBCHS), Yaoundé, Cameroon; 5 Department of Public Health, School of Health Sciences, Catholic University of Central Africa, Box 1110, Yaoundé, Cameroon; 6 School of Health Systems and Public Health, Faculty of Health Sciences, University of Pretoria Private Bag X323, Gezina, Pretoria, 0001, Pretoria, South Africa; 7 Institute of Medicine, Department of Public Health and Community Medicine (EPSO), University of Gothenburg, Box 414, SE - 405 30 Gothenburg, Sweden; 8 Faculty of Health Sciences, University of the Free State, Bloemfontein, South Africa

**Keywords:** Face presentation, pregnancy delivery, Cameroon

## Abstract

Face presentation is a rare obstetric event and most practitioners will go through their carriers without ever meeting one. Face presentation can be delivered vaginally only if the foetus is in the mentum anterior position. More than half of the cases of face presentation are delivered by caesarean section. Newborn infants with face presentation usually have severe facial oedema, facial bruising or ecchymosis. These syndromic facial features usually resolved within 24-48 hours.

## Introduction

Face presentation is a rare unanticipated obstetric event characterized by a longitudinal lie and full extension of the foetal head on the neck with the occiput against the upper back [1-3]. Face presentation occurs in 0.1-0.2% of deliveries [3-5] but is more common in black women and in multiparous women [5]. Studies have shown that 60 per cent of face presentations have one or more of the following risk factors: small fetus, large fetus, high parity, previous caesarean section (CS), contracted pelvis, fetopelvic disproportion, cord around the neck multiple pregnancy, hypertensive disorders of pregnancy, polyhydramnios, uterine or nuchal cord anomaly. But 40 per cent of face presentations occur with none of these factors [6, 7]. A vaginal birth at term is possible only if the fetus is in the mentum anterior position. More than half of cases of face presentation are delivered by caesarean section [4]. Newborn infants with face presentation usually have severe facial edema, facial bruising or ecchymosis [8]. Repeated vaginal examination to assess the presenting part and the progress of labor may lead to bruises in the face as well as damage to the eyes.

## Patient and observation

**Figure 1:**
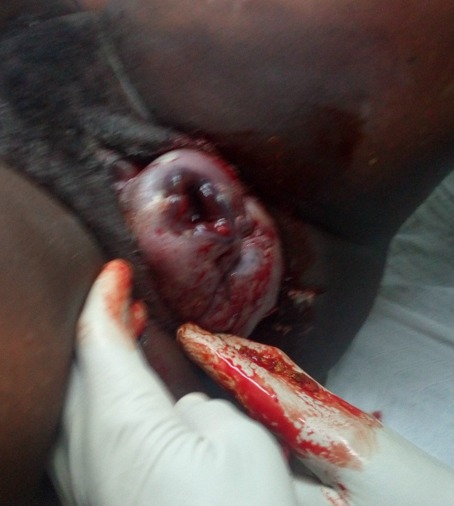
Men-tum anterior face presentation

**Figure 2:**
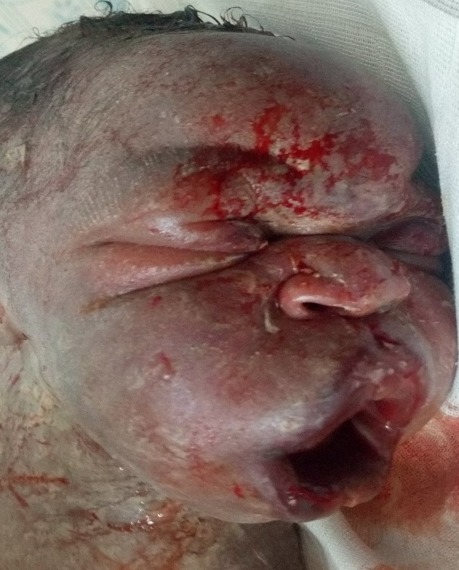
Bruising, marked lip and facial edema

**Figure 3:**
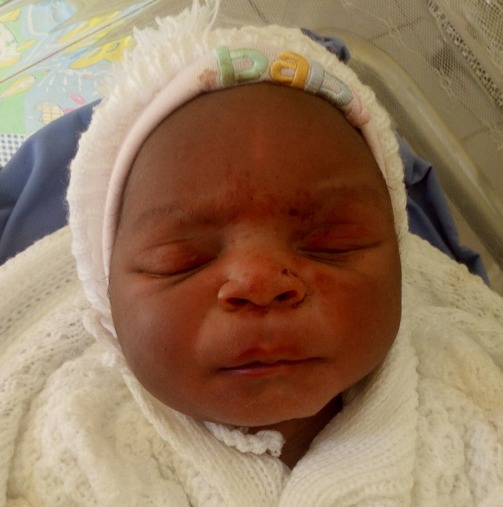
Baby 24 hours later with all syndromic facial features resolved

**Case presentation:** a 21 year old primigravida at 40 weeks gestation from the last normal menstrual period referred to our facility for prolonged second stage of labor after visiting two health centres. She labored for a total of 14hrs, membrane ruptured spontaneously 12hrs before referral. Amniotic fluid was documented by midwife to be clear. She attended antenatal clinics in Mbengwi health centre 5 times, was diagnosed of hepatitis B during antenatal consultations, received no treatment. She did not do any ultrasound due to financial constraints. On examination, she was healthy, in painful distress, blood pressure 131/76mmhg, pulse 85 beats/min, temperature 37.2^o^C SPO2 98%. On abdominal exams, uterus was gravid, fundal height 35cm, lie longitudinal, fetal heart rate 137bpm, cephalic presentation, engaged 2/5, with moderate contractions of 2 in 10 minutes. On vaginal examination, cervix was fully dilated, membranes ruptured, presenting part was face, mentum anterior. The conclusion made was mento-anterior face presentation (Figure 1). Paturient was counseled, labor was augmented with 1 amp of oxytocin in 500ml of glucose 5% and started at 10drops/mins. Ten minutes later she delivered a male baby with Apgar score 6/10, 8/10 on the first and fifth minute. The baby weighed 3.2kg, length was 50cm, and head circumference was 41cm. Syndromic facial appearance with marked edema at the baby's lips, face and scalp was evident and he had bruising on the right nasolabial groove and right cheeks (Figure 2). Physical examination of the infant's respiratory system, cardiovascular system, and his abdominal examination were normal, as was his neurological examination. Placenta was delivered by controlled cord traction 5mins later with all cotyledons. Delivery was complicated by a second degree perineal tear. Perineal tear was repaired with absorbable suture under local anaesthesia. Estimated blood lost was 350ml. baby received Hepatitis B immunoglobulins, hepatitis B vaccine and vitamin K were administered to the baby. 24 hours later, facial swellings resolved (Figure 3), baby breast feeds well. Baby and mother were discharged on day 3 postpartum all fine.

**Ethics**: informed consent: written informed consent was obtained from the patient's parents for the publication of this case report.

## Discussion

Face presentation is a rare obstetric event and most practitioner will go through their carriers without ever meeting one [3]. We presented a case of face presentation noticed in the delivery room on digital examination in a patient with no risk factors. In a poor resource setting as ours where ultrasound is not readily available, this event is often scary and confusing to most midwives and nurses. This may prompt repeated vaginal exams for confirmation of presentation. This intend will lead to bruising of the baby's face and delay effective management [8]. Exact knowledge about the fetal position and level is important for providing the correct management of this malpresentation. When face presentation is diagnosed, around 60% of cases are in the mentum anterior position, 25% are mentum posterior and 15% are mentum transverse [5]. The patient presented the most common form of face presentation (mentum anterior). Labor was augmented, vaginal delivery was attempted and successfully conducted. Facial bruising, lip and face edema are very common complication of face presentation. These complications usually resolve within 24-48 hours [9, 10] in this case facial edema completely resolved within 24hours (Figure 3) and baby breastfeed well.

## Conclusion

Repeated vaginal exams should be avoided when presenting part is unsure. Vaginal delivery should be attemped only on mentum anterior face presentation, in other cases, emergency ceserian section should be performed. Syndromic facial features in babies born from face presentation resolve completely within 24-48 hours.

## Competing interests

The authors declare no competing interests.
